# *In-vitro* renal epithelial cell infection reveals a viral kidney tropism as a potential mechanism for acute renal failure during Middle East Respiratory Syndrome (MERS) Coronavirus infection

**DOI:** 10.1186/1743-422X-10-359

**Published:** 2013-12-23

**Authors:** Isabella Eckerle, Marcel A Müller, Stephan Kallies, Daniel N Gotthardt, Christian Drosten

**Affiliations:** 1 Institute of Virology, University of Bonn Medical Centre, Sigmund-Freud-Strasse 25, Bonn 53127, Germany; 2 Department of Internal Medicine IV, University Hospital Heidelberg, Im Neuenheimer Feld 410, Heidelberg 69120, Germany

**Keywords:** Middle East Respiratory Syndrome, Acute renal failure, Human Coronavirus, Renal epithelial cells, Dipeptidyl-peptidase-4, Angiotensin-converting-enzyme-2

## Abstract

**Background:**

The Middle East Respiratory Syndrome Coronavirus (MERS-CoV) causes symptoms similar to Severe Acute Respiratory Syndrome Coronavirus (SARS-CoV), yet involving an additional component of acute renal failure (ARF) according to several published case reports. Impairment of the kidney is not typically seen in Coronavirus infections. The role of kidney infection in MERS is not understood.

**Findings:**

A systematic review of communicated and peer-reviewed case reports revealed differences in descriptions of kidney involvement in MERS versus SARS patients. In particular, ARF in MERS patients occurred considerably earlier after a median time to onset of 11 days (SD ±2,0 days) as opposed to 20 days for SARS, according to the literature. *In-situ* histological staining of the respective cellular receptors for MERS- and SARS-Coronavirus showed highly similar staining patterns with a focus of a receptor-specific signal in kidney epithelial cells. Comparative infection experiments with SARS- and MERS-CoV in primary human kidney cells versus primary human bronchial epithelial cells showed cytopathogenic infection only in kidney cells, and only if infected with MERS-CoV. Kidney epithelial cells produced almost 1000-fold more infectious MERS-CoV progeny than bronchial epithelial cells, while only a small difference was seen between cell types when infected with SARS-CoV.

**Conclusion:**

Epidemiological studies should analyze kidney impairment and its characteristics in MERS-CoV. Virus replication in the kidney with potential shedding in urine might constitute a way of transmission, and could explain untraceable transmission chains leading to new cases. Individual patients might benefit from early induction of renoprotective treatment.

## 

Coronaviruses (CoVs) cause human disease with symptoms ranging from mild respiratory symptoms to severe pneumonia [1]. In September 2012, a novel CoV termed the Middle East Respiratory Syndrome (MERS)-CoV emerged on the Arabian Peninsula with 163 laboratory-confirmed cases including 71 deaths so far (World Health Organization, December 2nd, http://www.who.int/csr/don/2013_12_02/en/index.html). Due to its distribution in several countries of the Arabian Peninsula there is a risk of global spread through travel and pilgrimage, posing a potential threat to global public health.

The clinical picture of MERS-CoV infection is characterized by acute atypical pneumonia and respiratory failure, resembling symptoms caused by SARS-CoV [2-5]. Acute renal failure (ARF) was described in a number of MERS cases, with potential influence on disease severity [2,3,5-7]. ARF has neither been a typical feature of SARS, nor has it been commonly observed in infections caused by any other CoV, including human HCoV-NL63, -229E, –OC43, and -HKU1. Of note, the majority (76%) of MERS-CoV patients were reportred to have underlying medical conditions such as diabetes, chronic cardiac disease and chronic renal disease [4,8].

It has been shown recently that MERS-CoV enters target cells not via the SARS-CoV receptor Angiotensin-converting-enzyme-2 (ACE-2), but via binding to Dipeptidyl-peptidase 4 (DPP-4) [9,10]. Both, ACE-2 and DPP-4 are expressed in several human tissues, including the kidney [11,12]. However, kidney infection has not been compared between MERS-CoV and SARS-CoV, and no post-mortem investigations on patients who succumbed to MERS-CoV have been done. To assess the potential role of kidney affection in MERS-CoV infection, we reviewed all peer-reviewed or communicated reports on MERS cases for information on kidney involvement. The search was performed in PubMed and Promed.Mail (http://www.promedmail.org) using the search term “coronavirus” on items published between 15th September 2012 and 16th September 2013. The broad search term was chosen due to inconsistencies of nomenclature before the consensus name MERS-CoV was announced [13].

In total, the search revealed 655 publications, including 508 articles in PubMed, and 147 reports in ProMed (Figure 1). All identified publications were reviewed in order to identify clinical descriptions of MERS-CoV infection that included information on kidney function, irrespective of whether they were pointing to normal or impaired function. In cases where two reports on the same patient were available, only the report with the more detailed clinical description was selected. From all identified reports, we extracted information on patient sex and age, country of patient origin and travel route, onset of illness, presence or absence of ARF, date of onset of ARF, day of illness on which ARF occurred, as well as clinical outcome (death or survival). Among 21 publications describing a total of 111 MERS-CoV patients (double-reporting of the same patients could not be excluded), a total of 7 publications were identified that specifically addressed kidney function [2,3,5-7,14,15]. In these 7 publications, a total of 12 MERS patients were described with disease onset dates ranging from April 2012 to March 2013 (Table 1). All patients were male with a mean age of 47 years (range 25–73 years), originating from Jordan, Saudi Arabia, and Qatar, including imported cases to the United Kingdom (UK), Germany and France. Nine of those 12 patients (75%) developed ARF during the acute course of MERS-CoV infection. For 6 of those 9 patients, dates of onset of ARF were available, suggesting a median time of 11 days (SD ±2,0 days) from first symptoms to onset of ARF. Six of 9 (67%) patients with ARF had a fatal outcome, while one of three patients without ARF died. However, the differences found for the fatality rates in cases with and without ARF during MERS-CoV infection were not statistically significant.

**Figure 1:**
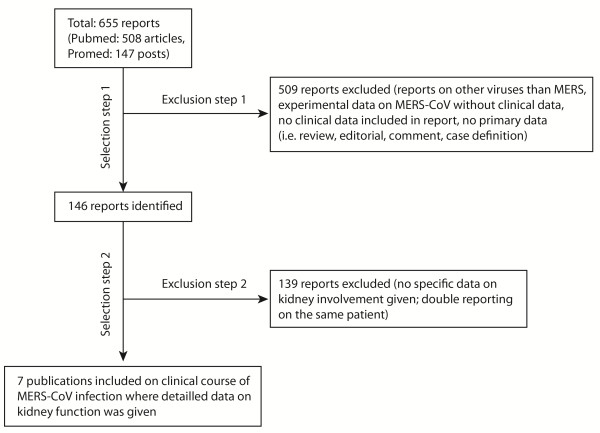
**Flowchart of study selection.** Systematic search strategies were applied to identify all reports on patients’ kidney function during MERS-CoV infection. Data sources used were Pubmed (http://www.ncbi.nlm.nih.gov/pubmed/) and Promed.Mail (http://www.promedmail.org) by searching the term “coronavirus” with a date range from 15th September 2012 (date of the first MERS-CoV report) to 16th September 2013. All identified publications were reviewed for clinical data on kidney function (irrespective of pointing to normal or impaired function). Data on patient sex and age, country of patient origin and travel route, onset of illness, presence or absence of ARF, date of onset of ARF, day of illness on which ARF occurred, as well as clinical outcome (death or survival) were extracted. In total, 7 publications were finally included that described a total of 12 MERS-CoV patients and their kidney function during the course of disease.

**Table 1:** MERS-CoV patients with explicit reports on presence or absence of acute renal failure (ARF)

**Nr.**	**Patient**	**Country**	**Onset of illness**	**ARF**	**Onset ARF**	**ARF on day of illness**	**Outcome**	**Reference**
1	25 y m	Jordan	Apr 12, day nd	Yes	nd	nd	Fatal	[7]
2	60 y m	Saudi Arabia	7 Jun 12	Yes	16 Jun 12	10	Fatal	[2]
3	49 y m	UK ex Qatar	3 Sep 12	Yes	14 Sep 12	12	Fatal	[15]
4	45 y m	Saudi Arabia	Nov 12, day nd	Yes	nd	nd	Recovered	[7]
5	45 y m	Germany ex Qatar	5 Oct 12	Yes	nd	nd	Recovered	[14]
6	70 y m	Saudi Arabia	5 Oct 12	Yes	16 Oct 12	12	Fatal	[3]
7	39 y m	Saudi Arabia	24 Oct 12	No	-		Fatal	[3]
8	16 y m	Saudi Arabia	3 Nov 12	No	-		Recovered	[3]
9	31 y m	Saudi Arabia	4 Nov 12	No	-		Recovered	[3]
10	64 y m	France ex Saudi Arabia	22 Apr 13	Yes	30 Apr 13	9	Fatal	[6]
11	51 y m	France	8 May 13	Yes	14 May 13	7	Recovered	[6]
12	73 y m	Germany ex Saudi Arabia	8 Mar 13	Yes	22 Mar 13	12	Fatal	[5]

y – years of age; m - male; nd - no data available.

To investigate a laboratory surrogate for susceptibility of the kidney to MERS-CoV in comparison to SARS-CoV, we analyzed cryoslides of a healthy human kidney for ACE-2 and DPP-4 expression by immunofluorescence. Staining using polyclonal goat-anti-human ACE-2 immunoglobulin (R&D systems) and polyclonal goat-anti-human DPP-4 immunoglobulin (R&D systems) showed that both receptors are abundantly expressed in the healthy human kidney, and both receptors are predominantly found in the epithelial layer of the renal ducts (Figure 2A), as well as in human primary epithelial cells derived from these ducts and in epithelial cells from the human airway tract (Figure 2B).

**Figure 2:**
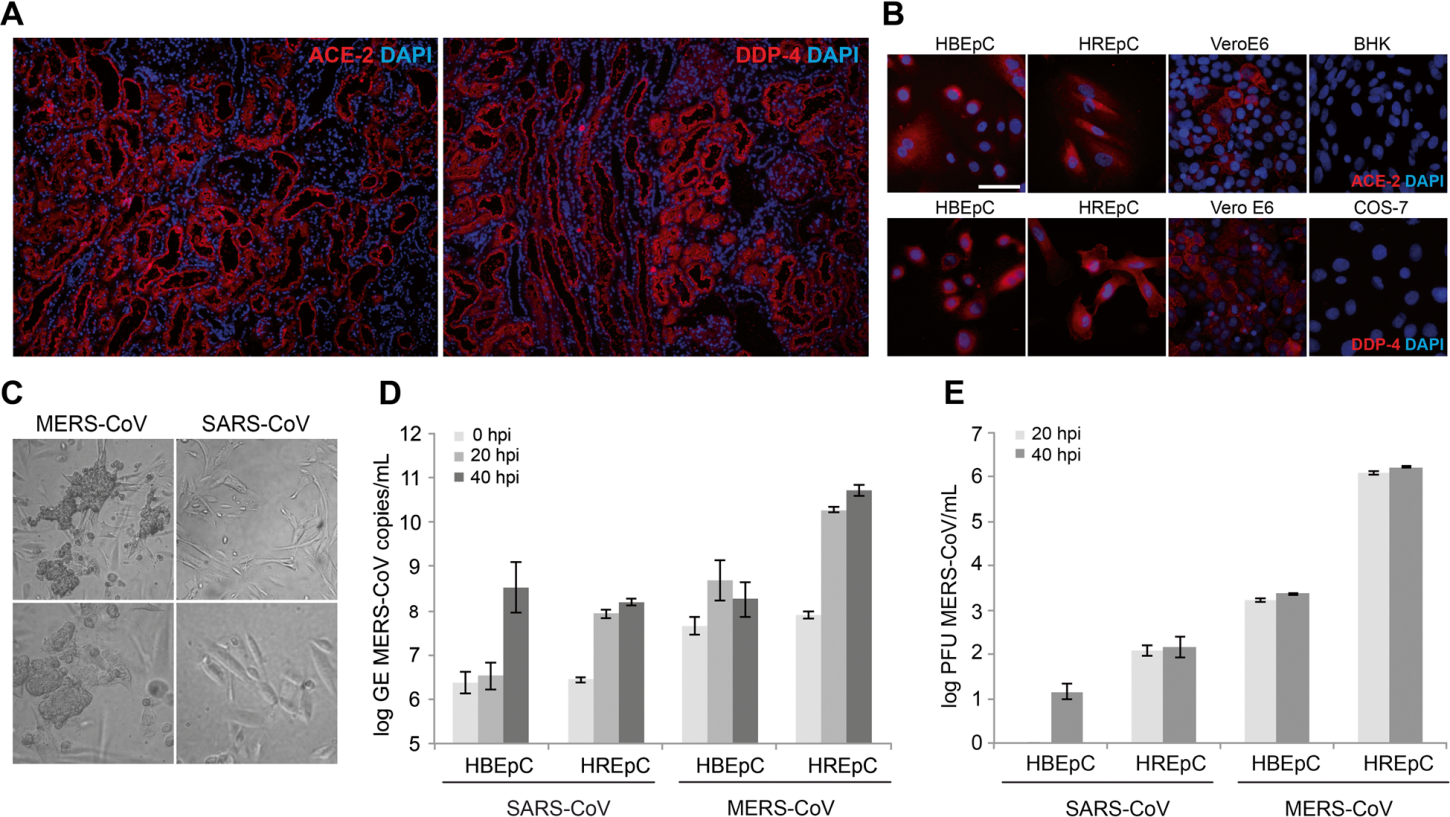
**SARS- and MERS-CoV receptor expression and virus infection experiments in human primary cells. (A)** SARS- and MERS-CoV receptor expression of human Angiotensin-converting-enzyme-2 (ACE-2) and Dipeptidyl-peptidase-4 (DPP-4), respectively, in cryosections of a healthy human kidney and **(B)** in primary bronchial (HBEpC) and renal (HREpC) epithelial cells. For positive controls, ACE2- and DPP-4-expressing primate cells (kidney cells from African green monkey [Vero E6]) were stained in parallel. Cell lines known to be negative for ACE-2 (kidney cells from Syrian hamster [BHK]) or DPP-4 (kidney cells from African green monkey [COS-7]) were used as negative controls. The white bar represents 50 μm. **(C)** Cell morphology and cytopathic effect (CPE) formation of human primary renal epithelial cells (HREpC) infected with MERS-CoV or SARS-CoV with 0.5 plaque-forming units of either virus per cell. A pronounced CPE formation 20 hours post infection (hpi) was seen only after infection with MERS-CoV in HREpC but not in cells infected with SARS-CoV. No CPE formation was seen in HBEpC infected with MERS-CoV or SARS-CoV (data not shown). Upper row: 100-fold magnification, lower row: 400-fold magnification, bright field microscopy **(D)** Replication of SARS- and MERS-CoV on HBEpC and HREpC determined by real time RT-PCR after 0, 20 and 40 hpi **(E)** Progeny virus measured by titration of supernatants in duplicates in a plaque assay in Vero cells. MERS-CoV replicates in HREpC with peak titers of 6.2 log plaque forming units (PFU)/mL, showing a 2,9-fold log difference between HREpC and HBEpC, while replication of SARS-CoV showed only a 1-fold log difference between bronchial and renal primary cells. Replication levels for each virus used are given as log of the genome equivalents (GEs) **(D)** or as plaque-forming units (PFUs) **(E)**. All virus infection experiments were performed in triplicates. Bars represent mean values, error bars represent standard deviation of triplicates.

To test whether kidney epithelial cells can actually be infected *in-vitro*, we performed comparative infection experiments with MERS- and SARS-CoV, using defined cultures of primary kidney epithelial cells (HREpC, Promocell, Heidelberg). As the bronchoalveolar epithelium of the lung constitutes the primary target compartment for both viruses, infection of human primary bronchial epithelial cells (HBEpC, Promocell, Heidelberg) was studied in parallel. As summarized in Figure 2, primary cells up to passage 4 were seeded at densities of 4x10^5^/mL and inoculated with either MERS-CoV strain EMC/2012, or SARS-CoV strain Frankfurt-1 at multiplicities of infection (MOI) of 0.5 for 1 hour. Cells were washed twice and supernatants were harvested immediately after washing, as well as 20 and 40 hours later. Cells were checked regularly for appearance of cytopathic effects (CPE) by light microscopy. Replication of both viruses was quantified by titration of supernatants in duplicate plaque assays on Vero cells as described previously, as well as by real-time RT-PCR [9,16].

Pronounced CPE consisting of visible and strong cell lysis occurred only in HREpC, and only upon infection with MERS-CoV (Figure 2C). Neither in HBEpCs upon MERS-CoV infection, nor in HBEpCs or HREpCs upon SARS-CoV infection any CPE was observed. Virus replication was nevertheless detectable by real time RT-PCR in all experiments, confirming successful infection (Figure 1D). The CPE in HREpCs consisted of rounding and detachment of cells, leading to cell death in the majority of cells already after 20 hours. Comparative quantification of infectious virus production between SARS- and MERS-CoV showed that MERS-CoV replicates to high titers in HREpC with almost 1000-fold higher concentrations of infectious MERS-CoV progeny than in HBEpCs. In SARS-CoV infection, only a small difference between primary kidney and bronchial epithelial cells was seen (Figure 1E).

Our results from *in-vitro* infection experiments suggest differences in kidney cell involvement might exist between SARS- and MERS-CoV infection. In a retrospective analysis of 536 SARS cases, only in 36 (6.7%) patients acute renal impairment was diagnosed. ARF occurred rather late in the course of disease after a median of 20 days from the appearance of first symptoms [17]. In our literature review of MERS cases, ARF occurred much earlier, namely in 6 of 9 sufficiently-described patients after a median of 11 days. To date, it is difficult to compare these clinical data with experiences from animal models. The only animal that exhibits clinical signs similar to human MERS-CoV infection is the rhesus macaque, but none of the available studies reported kidney failure in macaques [18-20]. Only very small amounts of viral RNA were detected in kidney tissue, and none in urine. Macaques might be of limited value for the study of MERS-CoV-specific kidney pathology as seen in humans.

While no autopsy data have become available for patients infected with MERS-CoV, post-mortem studies have been done systematically in SARS victims. Histopathological findings revealed mainly acute tubular necrosis without evidence of glomerular pathology, which is considered to be a consequence of systemic inflammatory response in the context of multi-organ failure, rather than a specific effect of viral infection of the kidney [17]. SARS-CoV has never been successfully isolated from *post mortem* kidney tissue of infected patients [21]. *In-vitro* studies of SARS-CoV with immortalized human proximal tubular epithelial cells showed replication without cell impairment as seen in our study, while no infection of podocyte cell lines and only low-level replication in glomerular mesangial cells (MC) was seen, providing further evidence against specific involvement of the renal tract in SARS-CoV infection [22]. In contrast, MERS-CoV was shown to efficiently replicate in a broad range of bat, primate and also human kidney epithelial cell lines that are commonly used as laboratory models [9]. The abundant expression of both viruses’ entry receptors in kidney epithelium argues against receptor-dependent limitations to viral kidney tropism. Specific interference of MERS-CoV with the induction of the interferon response provides one of many possible explanations for an increased capacity of MERS-CoV to replicate in kidney cells [23]. The clear differences in viral permissiveness of primary kidney epithelial cells suggest these cells to be appropriate models for the identification of host-specific restriction factors in the future. We are aware that cell culture resembles a limited model of viral infection which will not reflect the complexity of infection *in-vivo*. Nevertheless, our results have some congruence with clinical observations in a well-investigated MERS case [5]. Virus in the urine has been detected as late as day 12 of illness, albeit at low concentrations. Virus was detected in the urine but not in the blood, which could indicate autonomous virus replication in the kidneys. Of note, by using a model to assess the ability of CoVs to persist in the environment, it was shown that MERS-CoV has similarities to viruses that are transmitted via the fecal-oral route [24]. The authors suggest that this could hint for oral-urine transmission of MERS-CoV. Further data on virus shedding in the urine over the course of disease are urgently needed. If the kidney should indeed constitute a site of primary virus replication, shedding of virus in the urine might provide a possible source for human-to-human transmission, especially in health-care settings or among close family contacts [3,6,7]. Clinical guidelines should consider the possibility that MERS patients may benefit from early induction of renoprotective treatment.

## Competing interests

The authors declare that they have no competing interests.

## Authors’ contributions

IE, MAM designed the experiments, IE, MAM, SK performed experiments and analysis, IE, CD wrote the manuscript, IE, DNG, CD contributed to the final version of the manuscript. All authors read and approved the final manuscript.
